# “What’s happening in Syria even affects the rocks”: a qualitative study of the Syrian refugee experience accessing noncommunicable disease services in Jordan

**DOI:** 10.1186/s13031-019-0209-x

**Published:** 2019-06-13

**Authors:** Zahirah Zahrah McNatt, Patricia Elaine Freels, Hannah Chandler, Muhammad Fawad, Sandy Qarmout, Amani Saleh Al-Oraibi, Neil Boothby

**Affiliations:** 1 Columbia University Mailman School of Public Health, 60 Haven Avenue, B4, New York, NY 10032 USA; 2 Independent Researcher, Amman, Jordan; 3 University of Notre Dame, Amman, USA; 4 International Rescue Committee, Amman, Jordan

**Keywords:** Non-communicable disease, Health systems, Diabetes, Hypertension, Asthma, Refugees, Syria, Humanitarian response, Development, Jordan, Middle East and North Africa, Urban, Semi-urban

## Abstract

**Background:**

Humanitarian actors and host-countries in the Middle East and North Africa region are challenged with meeting the health needs of Syrian refugees and adjusting the response to contemporary humanitarian conditions – urban-based refugees, stressed host-country health systems and high NCD prevalence. Although several studies have explored NCD prevalence, utilization of services and barriers to access, these analyses took place prior to dramatic shifts in Jordanian health policy and did not account for nuances in health seeking and utilization behaviors or operational barriers. Accordingly, we aimed to understand the depth and nuances of Syrian refugees’ experiences accessing NCD services in urban and semi-urban settings in Jordan.

**Methods:**

A qualitative study was conducted to explore the healthcare experiences of Syrian refugees in Jordan. The study team conducted 68 in-depth interviews with Syrian refugees in urban and semi-urban locations in central and northern Jordan.

**Results:**

The findings indicated four themes key to understanding the healthcare experience: (1) emotional distress is a central concern and is frequently highlighted as the trigger for a non-communicable disease or its exacerbation; (2) service provision across all sectors – government, NGO, private – is complex, inadequate, expensive and fragmented, making engagement with the health sector physically and financially burdensome; (3) given financial constraints, participants make harmful decisions that further damage their health in order to reduce financial burdens, and (4) host-community members actively exhibit solidarity with their refugee neighbors and specifically do so during emergency health episodes. The findings from this study can be used to inform program design for forcibly displaced persons with NCDs and identify points of entry for effective interventions.

**Conclusions:**

Opportunities exist for humanitarian and host-country actors to provide more comprehensive NCD services and to improve the relevance and the quality of care provided to Syrian refugees in Jordan. Global and national funding will need to align with front-line realities and foster better coordination of services between host-country health systems, private actors and non-governmental organizations.

## Background

The Sustainable Development Goals (SDGs) put forth a vision of universal health coverage (UHC); and the commitment to “leave no one behind” calls us to consider the health concerns of the most marginalized, including refugees residing in low-and-middle income countries (LMICs) [1–3]. The forced migration of Syrians has been the primary example of difficulties faced when providing healthcare services to refugees, living in LMICs under challenging conditions, including growing numbers of refugees in urban settings, the enormous demands on host-country health systems and the demographic transition towards non-communicable diseases (NCDs) [4–6]. Over 5.5 million registered refugees have been displaced from Syria since 2011. The majority of refugees have settled in urban settings in Jordan, Lebanon and Turkey and face access challenges that differ from the camp-based experience [6–8]. This population, like others across the Middle East and North Africa (MENA), has a high prevalence of NCDs, ranging from 9 to 50% [5]. Seventy-four percent of regional mortality is due to NCDs including hypertension, diabetes, cardiovascular disease and chronic respiratory conditions [9–11]. In an attempt to meet the needs of this refugee population, a variety of interventions have been undertaken in the MENA region including, disease management algorithms, electronic medical records and the expansion of essential drug lists to include medications for NCDs [12, 13].

Despite these efforts, humanitarian actors remain overwhelmed by the number of refugees in need of NCD care, underfunded by global donors and fragmented in practice, resulting in responses that are ill equipped to meet contemporary demands [4, 6]. Host-country health systems are also challenged by the influx of refugees with chronic, long-term healthcare needs which may impact the quality and comprehensiveness of services provided [6, 14–17]. Jordan, for example, provided free services early on in the crisis, but over time, increased the cost of services making comprehensive care inaccessible for many [18]. In Lebanon, the health system was largely privatized, also resulting in high out-of-pocket expenses and access limitations [5, 6]. Early studies analyzed utilization patterns of Syrian refugees with NCDs and noted high utilization in public, private and NGO facilities. However, these analyses took place prior to dramatic shifts in Jordanian health policy and did not account for nuances in utilization behavior nor the quality of services being provided [7].

More recently, the UNHCR Health Access and Utilization Survey (2016) reported that 34% of Syrian refugees with hypertension and 37% of those with diabetes have been unable to access services and medications in Jordan [19]. In 2018, UNHCR reported that 2% of spontaneous returns from Jordan to Syria were a result of medical needs and the high cost of treatment in Jordan [20]. Additional literature described well-known barriers to access, including cost of services, medications and transportation [5, 9, 10, 21]. However, little documentation exists about the personal experiences of refugees, the operational barriers they face when accessing services, and how they make decisions amidst a myriad of interconnected life pressures. Moreover, the majority of studies to date have been quantitative and unable to capture refugee perspectives, document the complex nature of patient encounters, and identify refugee-driven solutions. The inattention to refugees’ own perspectives is unfortunate and likely contributes to ongoing invisibility and absence from local, national and global decision-making.

Accordingly, we aimed to understand the healthcare experiences of Syrian refugees residing in Jordan in 2018, with specific attention to personal perspectives about illness, approaches to healthcare decision-making, operational barriers faced when accessing care, reflections on healthcare quality and methods for coping with challenges of health care access. Jordan provided a relevant setting for this study because the country is middle-income and located in MENA, a region that has undergone a demographic transition resulting in high rates of NCD deaths [4, 7]. Jordan also hosts over 650,000 registered Syrian refugees (some estimates including unregistered persons are as high as 1.4 million) [22]. In the early stages of the crisis, Jordan made select primary care services free for Syrian refugees. However, in January 2018, Jordan increased the cost of services for Syrian refugees to the “foreigner rate,” with 80% of fees paid prior to services being rendered [7, 23]. Opportunities exist to better understand the Syrian refugee experience accessing NCD services with the aim of ensuring high-quality, continuous, coordinated and comprehensive care.

## Methods

### Study design and sample

A qualitative study was conducted to explore the healthcare experiences of Syrian refugees in Jordan. The study team conducted in-depth interviews with Syrian refugees in urban and semi-urban locations in central and northern Jordan. Participants resided in apartments, houses and in rare cases tents, and lived with their immediate family or shared housing with other families. These locations were selected for two reasons, (1) the majority of Syrian refugees reside in the northern and central governorates and (2) our humanitarian partner, International Rescue Committee (IRC), provided services in these areas. The sample included adult refugees over the age of 18; who lived in urban/semi-urban settings in Jordan; had at least one of three diagnoses - diabetes, hypertension or asthma; and sought care from one of two static IRC clinics in 2016, but did not return for care. People who did not meet these criteria were excluded. Participants were purposively sampled [24] to include a balance of men and women and a range of ages. Interviews were conducted until theoretical saturation was achieved [25], the point at which no new concepts arose (*n* = 68).

### Partnership

This study was a collaborative effort between the Columbia Global Centers | Amman, Columbia University, Department of Population and Family Health at Mailman School of Public Health and the International Rescue Committee in Jordan. The study team from Columbia developed the study design and oversaw implementation of the work. The team from International Rescue Committee advised on the research question and the study design. They also identified potential study participants and obtained the first verbal consent prior to any communication from Columbia researchers. This collaboration created a platform for conducting research that was both useful and relevant to humanitarian practitioners in Jordan.

### Tool development and data collection

In-depth interviews were conducted by Jordanian interviewers in Levantine Arabic during the month of April 2018. Data collectors utilized a 10-question interview guide (Appendix 1) that investigated several topics related to the healthcare experience including (1) perceptions of own illness (2) access and barriers to health services, (3) management of illness, (4) use of medications and (5) decision-making and approaches to coping with illness. The interview guide was developed by the lead author, reviewed by two senior researchers at Columbia University then modified and translated by Arabic speaking co-authors and a professional translator in Jordan. The interview questions were chosen in order to capture experiences and perspectives before, during and after healthcare encounters. The interview guide was modified when necessary during the data collection phase, based upon team reflections and themes that arose from the data [26].

All interviews were held in the homes of participants to increase the likelihood of comfort and confidentiality and, on average, were 45 min in duration. Interviews were audio recorded with permission of participants. When participants declined recording, detailed written notes were taken. Following each interview, data collectors completed field notes to document key aspects of the interview experience and interview content. The full research team, guided by the lead author, held reflective sessions 2–3 times per week during the data collection period. Reflective activities aimed to document themes arising in the data, consider modifications to the interview guide, address biases and adjust interview approaches to improve interview quality [26]. All notes and recordings were stored on 3 password-protected laptops, while still in the field. Materials were then transferred to 1 password-protected laptop locked in an office at the Columbia Global Center in Amman. Recordings were uploaded to a secure and encrypted portal for transcription by a professional transcriptionist.

### Researcher selection, training and orientation

The full study team included a mix of national and international researchers from diverse backgrounds in pharmacy, forced migration, health systems, public health and urban planning. Researchers participated in a 3-day training and orientation in Amman, Jordan focused on qualitative methods, confidentiality, informed consent and interview skill-building. The training schedule included extensive time allotted for practicing the interviews together and also with persons unrelated to the project. The interview team included 3 women, AO, SQ, NA and was led by ZM. Data collectors were recruited through the Columbia Global Center in Amman. Three team members either had a graduate degree or were in pursuit of a graduate degree. One team member held a bachelor’s degree and was a practicing pharmacist in Jordan.

### Ethics

All research procedures were approved by the Institutional Review Board at Columbia University Medical Center in New York, USA, the Syrian Refugee Affairs Directorate in the Jordanian Ministry of Interior and the Institutional Review Board at King Hussein Cancer Center in Amman, Jordan. This study was implemented in partnership with IRC. IRC’s role was to inform the study design and identify potential participants. Utilizing the IRC database, a staff member identified all patients that met the study criteria and had full data available about their demographics. Two hundred ninety-seven people met the criteria. This list was randomized and an IRC staff member called potential participants to notify them that they qualified for the study and to gauge their interest. Though the sample was randomized, participants were purposively sampled to ensure a balance of men and women and a range of ages. A research team member then followed up, introduced herself, described the study goals and objectives, gained the first verbal consent to participate and scheduled the interview. Nine people declined participation, either because they reported not having an NCD or they were unavailable during work hours.

A second written consent was obtained in-person before the interview began. The participants were read a standard consent form that provided information about the study and contact information should problems arise. All respondents gave verbal informed consent by telephone followed by written informed consent in-person. Names and contact information were recorded during phone conversations but kept separate from data and destroyed after the interview. Upon agreeing to participate in the study, an identification number was assigned to each participant. The data was de-identified for the analysis phase and the reporting of findings. Interviewers were trained as described above but also received a brief introduction to psychological first aid (PFA). PFA skills were not needed during the data collection period but prepared the team members to manage urgent needs. Each interviewer also had referral information for mental health, education, physical health, livelihoods and other humanitarian aid related services. Organization names and contact information were given to participants when the specific subject matter was relevant to their needs. As required by the local IRB, participants were compensated $10 Jordanian Dinars ($14USD) to account for their time and their provision of coffee, tea and biscuits to interviewers (a common custom for welcoming guests).

### Data analysis

Recorded interviews were transcribed and translated from Arabic to English by a professional transcriptionist. This transcriptionist previously worked with the team leader to transcribe interviews from the same locations in Jordan. The data collection and analysis phases were guided by the qualitative principles of thematic analysis and the constant comparative method [27] in which interviews were coded inductively and themes were identified and compared across the dataset. A preliminary thematic structure was created based on open-coding of a sub-sample of 15 transcripts read by 5 researchers who initially independently developed themes. The study team was expanded to include master’s trained public health practitioners, to support extensive coding and bring added diversity to the analysis process. A comprehensive codebook was created and all transcripts were analyzed using Dedoose (version 7.6.21). Efforts were made to present representative narratives from across the dataset. Where an uncommon narrative was reported, the number of participants sharing this perspective were noted. Rare, but powerful narratives, were occasionally presented as they exhibited the depth and gravity of a specific experience. Where feasible, the study methods and reporting have been completed in accordance with the Consolidated Criteria for Reporting Qualitative Research [28].

## Results

Interviews were conducted with 68 respondents (Table 1), all of whom were Syrian refugees between the ages of 18–59, residing in and around three governorates (Irbid, Mafraq, Amman) in northern and central Jordan. 50% of the respondents were women, 48.5% had diabetes, 72.1% had hypertension and 19.1% had asthma. 92.6% had comorbid conditions including, but not limited to epilepsy, gout, kidney disease, cancer, heart disease, stroke, umbilical hernia, depression, obsessive compulsive disorder, thyroid disease and hypercholesterolemia. 27.9% had disc disorders, due to injury or degenerative disc disease. 97% of participants visited 3 or more facilities in order to access primary care, secondary care, laboratory testing and medications. 41.2% of participants visited 5 or more facilities in order to access the above services. These institutions included, but were not limited to, NGOs (i.e., International Rescue Committee, Médecins Sans Frontières, Doctors of the World, International Federation of Red Cross and Red Crescent Societies), government facilities (i.e., Al-Ramtha hospital), UNHCR supported facilities (i.e., Jordan Health Aid Society, Caritas) and private clinics.

**Table 1:** Participant demographics

	*N* (%)
Total	68 (100%)
*Women*	34 (50.0%)
*Men*	34 (50.0%)
NCD diagnoses	
*Diabetes*	33 (48.5%)
*Hypertension*	49 (72.1%)
*Asthma*	13 (19.1%)
*Comorbidities*	63 (92.6%)
Residence	
*Mafraq*	24 (35.3%)
*Irbid*	43 (63.2%)
*Amman*	1 (1.5%)
Age	
*18–29*	2 (2.9%)
*30–39*	13 (19.1%)
*40–49*	24 (35.3%)
*50–59*	29 (42.6%)
Other NCD diagnoses	
*Mental illness*	9 (13.2%)
*Disc disease/injury*	19 (27.9%)

Names of NGOs were de-identified in order to present systems challenges with objectivity. Interview excerpts were presented as examples of thematic areas and were labeled by gender, disease diagnosis and participant’s study identification code. Forty individual respondents were quoted in the findings and four of those respondents were quoted twice.

We identified four themes as central to understanding Syrian refugee experiences accessing NCD care in Jordan. These learnings are the most prominent and are highlighted as opportunities for program improvement and policy development.

1. Emotional distress is a central concern and is frequently highlighted as the trigger for a non-communicable disease or its exacerbation.
2. Service provision across all sectors – government, NGO, private – is complex, inadequate, expensive and fragmented, making it physically and financially burdensome to engage with the health sector.
3. Given financial constraints, refugees make harmful health decisions in order to reduce financial burdens on themselves and their families.
4. Host-community members actively exhibit solidarity with their refugee neighbors and specifically do so during urgent or emergency health episodes.

### Emotional distress and physical health

Psychosocial wellbeing and emotional distress were central concerns and directly linked to the onset or exacerbation of non-communicable diseases. In many cases, respondents prioritized emotional distress as the reason for their illness, dismissing diet, exercise and other common risk factors. A variety of reasons for emotional distress were described, including missing separated family members, worrying about debt and the inability to meet basic needs (including food and water). Others reflected on the absence of activity – work and play – and its negative impact on the wellbeing of adults, children and communities. Several respondents described the connection between their physical health and their emotional state,

*‘It will get better if God manages to [bring] me, my boys, my girls, my husband and my house all together. That will better the mentality. As you know we [are] in a foreign land … We left our home and business and came here. Sometimes we cannot even get a bottle of water to drink. My kids are doing their best. We are paying the house rent, electricity, water and transportation costs. My young son is studying Tawjihi [secondary school exams]. He wanted private lessons but I don’t have enough to give him for that. [Who] wouldn’t get blood pressure problems? My husband has been in Syria for 5 years … He isn’t able to enter … If I hear bad news my blood pressure will increase. We put ourselves between God’s hands, he created us and he knows what to do with us.’* (woman with hypertension, 517).

*‘I pay the 4 dinars so I get my medication. I have a Jordanian neighbor; I get the money from her … What can I do? You tell me. I mean, we don’t have much to eat. I don’t have a refrigerator. I doubt that you will trust me if I tell you that my sons go to school on empty stomachs.’* (woman with diabetes, hypertension, asthma, 502).

*‘Sometimes I wouldn’t have eaten and it would reach 300, and other times I would have eaten and it would be in the evening, and the reading would be 160 or 200. It’s because of sadness – I don’t believe in any of this stuff, but sadness is the reason behind all illnesses.’* (woman with diabetes, 415).

Other participants spoke of how stress and anxiety about current and past family tragedies made it difficult or impossible to achieve physical wellness. Respondents consistently linked these and other emotional pressures to their health outcomes, as well as their inability, even when adhering to medication regimens, to lower blood sugar and blood pressure levels or generally maintain good health. Several respondents articulated their feelings of sadness and shock, guilt and anxiety, and how these feelings influenced their perceptions of wellness,

*‘We only had one person in our area [in Syria] with this illness – [my] uncle’s wife. We always thought it was a dangerous disease. We’d ask what is this blood pressure that she keeps taking medication for? But here, even the 20-year-old people get it … Just yesterday, two of my cousins died … Just yesterday we heard about it. So, how will we not get all these illnesses? --- What’s happening in Syria even affects the rocks.’* (woman with hypertension, 604)

*‘He said I should [eat a] diet [good] for diabetes. I already know this information. I know what I should eat … For diabetes patients, diet and food is not important. The mood and mentality play [a] big role. For instance, [if] I receive a call from Damascus [in Syria], I feel sad for one month. I am the only one who left the prison… My three brothers are there. My mother is on her [own]. Diabetes cannot be stable as long as my family is living like that.’* (man with diabetes, 408).

*‘[My] diabetes level was 500. My blood pressure was high and my condition was bad. They treated me here and sent me to the hospital. I get upset when I know about the situation with my children. Sometimes there are raids and shooting [in Syria]. I was very nervous when my daughter got attacked by two raids and her house destroyed. Her daughter of three months was in her arms. Her husband, son and she were under the collapsed house. The rescue crew came. They saw the baby crying and covered with blood. They knew there were people under. They took the child and rescued my daughter. Her husband broke his back. Her son was hit in the head. And she was in critical condition. How can I not be upset?… Being upset, this is what makes diabetes and blood pressure worse.’* (woman with diabetes, hypertension, 416).

Some of the psychosocial concerns expressed could be mitigated by improved financial conditions, reunification with family members or other social solutions. Other expressions of distress appeared to require clinical support from a mental health professional. However, it is unclear whether or not mental health needs were being well recognized at the primary care level. Additionally, a small fraction of participants were referred to mental health services but declined to attend. One participant articulated his frustration,

*‘I know about [services at NGO 1]. They have a lawyer there and a psychiatrist … not a psychiatrist, just a counsellor. The counsellor asks us what kind of problems we have. I mean what kind of question is that? Our problems are clear. He tells us not to get angry. Well, is it a choice to be angry? No, it isn’t.’* (man with diabetes, 623).

Only 9 participants disclosed obtaining services from a mental health provider. Of these, eight were either neutral about their care or satisfied with their care and described seeing reductions in symptoms. Two respondents noted,

*‘Respondent: Yes, they called me and I went and attended two sessions [for counseling]. I also had one this week. She calls me on the phone and tells me to come and talk. Interviewer: So, you feel comfortable with her to talk to her? Respondent: Yes, we talk to one another and she asks me stuff – You know, one speaks and lets things off of their chest.’* (man with diabetes, hypertension, 425).

*‘Just once I got sick with Obsessive Compulsive Disorder. I got sick and I stayed sick for almost a year. I was treated in a place called [NGO 4]. I was treated by a psychiatrist. I remained for almost one year taking the medicine. I only wanted to get better. I couldn’t sleep for ten days. I couldn’t sleep at all. I used to see bad things. I just wanted to be cured because I couldn’t sleep. I couldn’t sleep at all at night. For example, when I prayed, I saw bad things while praying. You know? I would repeat the prayer. And the same thing again. I cried during the day … I was very depressed. The important thing was that I was treated, and I went, [back to] normal.’* (woman with hypertension, 403).

Overall, respondents consistently expressed emotional distress and linked it to their inability to control symptoms of their diabetes, hypertension, and to a lesser degree, asthma. Several signs of distress could be improved through social and emotional support from friends, family members and the surrounding community. However, significant distress was associated with past or present violence against family members who were unable to escape Syria.

While few options exist to solve the current issues of violence, those residing in Jordan may need more comprehensive, accessible and relevant psychosocial interventions and better integration of those services with other parts of the health sector (i.e., primary healthcare). A substantial number of participants needed more specialized mental health services but had not been recognized or referred by their primary care provider, highlighting a gap in service provision. Opportunities exist to better recognize and respond to the connections between physical and mental health.

### Service provision is complex, fragmented, inadequate and burdensome

#### Government, NGO, private

##### Government

Syrian refugees in Jordan utilize a mix of providers in an attempt to access comprehensive services for NCDs, including government, NGO and private facilities. In 2014, and again in 2018, Jordan enacted health policies that increased the price of services for Syrian refugees, making care in the public sector largely inaccessible. After the policy changes, most participants avoided government facilities because cost was a significant barrier. One man described how the policy changes influenced his choices about where to seek care, saying,

*‘I used to go there and they gave me all the needed medicines. They were very nice. It was totally free … [They] ceased covering people who go to government hospitals and people started to talk about [NGO clinics]. Hence, I started to go there.’* (man with diabetes, hypertension, 615).

*‘Only God knows what will happen. You know our situation. We cannot go to [the government] hospital even if we want to. I am telling you, I wanted to go … I had to get tests done. I did not have the money. We need twenty dinars and ten for medication. How can we find those?’* (woman with diabetes, 622).

Others stated that when they did go to government facilities, it was only for emergency hospital care. Often the first encounter was for severe pain and to seek a definitive diagnosis for those symptoms. Upon receiving a diagnosis, the patient would then seek treatment from the same facility for health concerns, such as cardiac catheterization or kidney stone removal. However, numerous participants described the financial barriers to receiving secondary or tertiary care, barriers that resulted in them being unable to undergo important procedures. These two respondents were diagnosed with diabetes and/or hypertension, but here explained their failed attempts to get health services for their comorbidities,

*‘I can’t afford it [cardiac catheterization]. It costs 400 dinars [at a government facility], and I don’t have 400 dinars. I would have done it a long time ago. But we don’t have it. We have the house rent, and you know, what are you going to do about it? You know the situation of the refugees.’* (man with diabetes, hypertension, 503).

*‘When I took him to [a] special doctor he said that he had [a] big kidney stone. He needed to remove it by surgery. It is what was causing [his] fainting. [They] took him to [a government hospital]. They gave him pain killers and sent him back. He feels the same pain at night. I wanted to see a doctor to understand what he has. After taking him, he said that he needs a surgery right away. I asked about it and they said it is expensive. I do not even have the bus fee! People told us to offer our documents to [NGO 7].’*

(man with hypertension, 414)

Some respondents were faced with the high cost of secondary or tertiary care in the government setting but then sought help from NGOs, UN agencies and other private donors. In these cases, most recalled long hospital waiting lists for surgeries or long waiting periods to receive financial aid. Two men with diabetes highlighted their concerns,

*‘Operations are few here in ___ and take a lot of time when you register. For example, gallbladder operations take four months. There’s no one to do it even if you want to pay the fees. It costs about 400-500 dinars. We don’t have 40 dinars, so imagine paying 400. You may wait for … four months till you do it or even till a donor comes, as they give us a turn in [NGO 8] … They scrapped the subsidy from us, us Syrians. We [went to] the government hospital … Now this isn’t available. The examination fee was one pound and 65 piasters. We pay now 16 dinars.’* (man with diabetes, 402).

*‘In 2014 … when I went to the doctor there, he told me that I had an umbilical hernia and that it is not a big thing. I still have severe pain and it is becoming bigger. The last time he told me that my umbilical hernia became big and I need to do a surgery to remove it. When I went to the [government] hospital, they asked for 200–400 dinars … I went to UNHCR and explained … my situation. I call them once in a while and they tell me that my request is still under consideration.’* (man with diabetes, 614).

No one utilized government services for primary care or basic healthcare needs. These needs were either met through visits to NGO clinics, direct care-seeking from pharmacies or went unmet as a result of various barriers. The sole reason cited for not attending government facilities was cost. As a result government services were only utilized for emergencies or for needs that could not be met at NGO clinics. The government policies instituted in 2014 and 2018 continue to push patients out of the public sector and into NGO facilities (when convenient and available) and private institutions (when financially viable). These findings shed light on the shift in care-seeking behavior from the utilization of a mix of service providers to a focus on seeking care from NGOs. It also presents opportunities for better integration of government and NGO services in order to reduce the burden on both sectors and on their most vulnerable patients.

#### Non-governmental organizations

Many refugees, particularly those residing in northern Jordan, visited NGO clinics for basic primary care services. Providers listed by participants have included the International Rescue Committee (IRC), Jordan Health Aid Society (JHAS), Caritas, Médecins Sans Frontières (MSF), Al-Emirati Hospital, Doctors of the World, the International Federation of Red Cross and Red Crescent Societies and several others. These organizations have served as an important source of care for this population. Gratitude was frequently expressed in relationship to NGO services, noting several practices that were satisfying to patients and occurred in some NGO clinics but not the majority. The first was appointment scheduling. Respondents noted that scheduling appointments for a specific time made their visits less burdensome. They also lamented that this practice only occurred at one NGO and that others required them to arrive early on the specified day and wait hours for service. The second practice was the distribution of blood pressure and blood sugar monitors for use in the home. Both practices encouraged better self-management, with respondents stating,

*‘At first, I went to [NGO 1]. Because at a particular time, the [NGO 2] ceased and I needed my medicines, so I went to [NGO 1] and they helped me a lot. But the problem with [NGO 1] is that there are no appointments. So, you wait all the day long for your turn … [On the] contrary, [NGO 2] has a good appointment system - they give the hour and minute. Even if you will be late for their appointment, you just call them and tell them that and they will give you another appointment.’* (man with hypertension, 515).

*‘They [NGO 2] always send me a letter. Every month they send me a letter to tell me of my appointment. They even write the date and the exact hour.’* (woman with diabetes, hypertension, 510)

*‘At first, they gave us instructions for using the device [blood pressure monitor]. We used it [at home] and they came again. They told me to show them how I use it. I showed them and they said okay. They come here every time. Things are good.’*

(man with diabetes, hypertension, 411)

A third practice that improved the satisfaction of patients were home visits, made in order to provide services to patients who had difficulty visiting a clinic. This service was particularly lauded by elders in the community, their adult children who provided informal homecare and persons living with physical disabilities. Respondents praised this practice, focusing on the convenience and the frequency of visits,

*‘I personally have my father who is physically disabled and they come to his house and visit him, check [on] him and give him the medicines. He had some blisters [on] his legs and they … offered him the medicines needed. Praise God.’* (man with diabetes, asthma, 513).

*‘I came here and there was complete care, I take my medication from [NGO 2] … They gave me a blood pressure device, a diabetes device and they come to treat me at my home. The ladies there are intelligent and nice. They give me all of my medication, visit me every 15 days and they even give me two Zain phone cards in case I need to call them for anything.’* (woman with diabetes, hypertension, 619).

The above services were emphasized by participants as important to their patient experience and as either a factor that increased access or increased self-efficacy. While there was appreciation for the care provided by NGOs, many specific concerns arose as participants described their barriers to receiving comprehensive care for their NCDs, or other ailments. NGO facilities are the most financially accessible but are often only funded for limited services (i.e., select NCDs, solely women’s health or basic primary care). In addition, NGOs face cuts in funding that suddenly make select medications unavailable in their pharmacies. The most common concern expressed was the inability to access specialists (i.e., nephrologists, urologists, dermatologists, endocrinologists), secondary services, and advanced laboratory testing in NGO facilities. Participants also noted frustration with being unable to get dental care or visit an ophthalmologist or optometrist. Dozens expressed frustration about the narrow selection of services available,

*‘Indeed, I went to the [NGO 2]. They told me they don’t cover triglycerides and cholesterol health issues. They asked me to give them my mobile number so as to call me if they start to cover them. Since then, it [has been] eight months, no one called me … Until this very moment, they don’t offer all the examinations needed. For example, if you need thyroid gland, hypothalamus, or any other similar examination then you need to pay for it from your own money [in the private or government facility].’* (man with diabetes, 614)

*‘We are living in sorrow. Can you see this [pointing to her teeth]? How can I fix them and how much will they cost? My tooth and face were swollen and I went to them … I went to the doctor and he gave me a paper with an appointment after two months to treat my tooth. I waited for a day or two and my tooth was swollen … He wanted me to wait 2 months to pull it out or fill it … How can I wait with a swollen tooth without treatment, and so I did not go back to them, let them all fall [out] … How would I feel? What to feel other than despair and regret for the life we lost and fear of what is coming?’* (woman with diabetes, hypertension, 407).

Medication shortages were also a concern to this population. Most complaints were about stock outs in facilities (medications that were once available but occasionally could not be found). A related concern was the absence of specific medications that were never available and always had to be sourced in the private sector. Both scenarios negatively impacted the quality of care provided, by introducing extended periods where patients were not able to adhere to medication regimens. These respondents described accessing primary care in the NGO setting but being unable to get medications and deciding to lower dosages or wait to be notified that the medication was back in stock.

*‘*Respondent: *At first, I used to go and take all my needed medicines, but they started to tell me that they don’t have medicines in the last three months. Interviewer: Do you buy the medicines from the [private] pharmacy? Respondent: No, I can’t. I don’t have money. I borrow [medicines] from people. Worst-case scenario, when I don’t have money and can’t find someone to give me the medicines, I start taking lower dosages … so they can last longer.’* (man with diabetes, hypertension, 506).

*‘They do not have insulin. I went to get insulin for my wife. They said they do not have it. They are waiting for donations. They are doing what they can. There is no insulin now. They are waiting.’* (man with diabetes, hypertension, 611).

As a result of the narrow selection of services available in NGO facilities and the high cost of care in government settings, participants must navigate a complex system and visit 3 or more facilities in an attempt to create a comprehensive healthcare experience - visiting 1 location to see a physician, another for laboratory testing, a third for medications that were unavailable in setting 1 or 2, and possibly a fourth or fifth for any ailment not treated in the prior settings. Many participants expressed the sense that seeking care was burdensome, not only financially but physically and emotionally.

*‘I went to [NGO 8] when I first came here, then after that I went to [NGO 5]. Then I went to [NGO 1] for a bit. I had a file, a diagnosis, every time the doctor sees me he prescribes a medication… Then it became crowded… Then [NGO 2] opened, and I told them I wanted to go there just for this illness, and I did. They chose me and started treatment with pills at first, but they observed that it wasn’t working. Then I went to ___ to see [a private endocrinologist]. She gave me pills there … it cost 40 dinars … I bought it the first month, then my neighbors bought it for me the second one, then it didn’t work out after that. I went back to [NGO 2] … I told them about the medication I was taking and that it cost 40 dinars, and then asked them if they could help me with it. They told me they couldn’t, but they had an alternative for it, which was insulin, but I didn’t want to take insulin. After that I went to [NGO 4] and they told me to get a medical report from the Ministry of Health … They [said] that I didn’t need this treatment and medication, so they referred it to the commission. The commission got it, refused and suggested alternatives, which didn’t work, so I was forced to go back to insulin.’* (woman with diabetes, 418).

*‘They told us about this urologist in [NGO 3], and we went to him. He did the imaging and told me that I had a cyst above my left kidney … and had an enlarged prostate. I asked him “Is it cancer?” but he said “No, where are you getting this from?” He said that it was benign and that the doctor will write me a treatment. So, we went to [NGO 3], which is a long trip, and it was really hot. As you know, ___ is a hot place, and sometimes you might go there and not find your medication. So, we told [NGO 2] – We had medical reports from [NGO 3] that I showed them, and they told me to go get my medication from [NGO 1]. So, I ended up taking my prostate and triglyceride medications from [NGO 1]. [NGO 2] didn’t have it.’* (man with hypertension, 618).

Most respondents described additional burdens including having to incur travel costs that outweighed the benefit of visiting a free clinic or having informal work that made clinic visits challenging. Additionally, participants described long wait times in facilities and occasionally being turned away from a clinic that did not have the capacity to serve them. While utilization of health services was generally high among this population, financial barriers and operational weaknesses resulted in disjointed care and some participants making decisions to cease care.

*‘It [the clinic] is far and I am an old woman. It is hard for me. Buying it [medication] from here [nearby pharmacy] is more comfortable for me.’* (woman with hypertension, 625).

*‘You go to [NGO 1]. If you’re not there by 6 o’clock, at 8 the queue is no more. Yesterday, my sister-in-law and my cousin went there at quarter to 8, and they told them there were no more turns for that day … What should I do, take a mat and sleep there? I swear to God that’s what’s happening to us.’* (woman with hypertension, 604).

*‘They were giving me medicine … I ran out of it, so I have to buy it from [the] pharmacy. I cannot go to [the clinic frequently] because I work … So, I have to buy medicine from [the] pharmacy … I am planning on taking a day off next week to get it [from NGO 1] [because] it is expensive [at the pharmacy]. I pay 7 dinars for two boxes. It is impossible for me to go to a doctor outside [NGO 1] or [NGO 2]. My financial condition does not support that.’* (woman with hypertension, 612).

Finally, each NGO had their own criteria for determining who qualified for their services. These criteria, though helpful for humanitarian actors, created confusion among potential patients and resulted in missed opportunities to provide care for communities on the margins. In some cases, these criteria were factual in nature (i.e., services only being provided to widows), in other cases, conversations between patients and staff led to misunderstandings about formal procedures (i.e., number of visits allotted per month).

*‘I went to [NGO 6] and she told me if I am not registered there, I cannot take the medication. I asked her how can I register and she said I have to be a widow or divorced. I told her that we are not divorced but she asked for the divorce [papers] which I don’t have and so I would have to pay for it at the same price as [an] outside [pharmacy]. So, I stayed two days without taking it [hypertension medicine] until I [went] to [NGO 3].’* (woman with hypertension, 518).

*‘[NGO 3] has one issue – it is about the number of visits we are allowed. For example, if I go today and they examine me for free, I am not allowed to get checked and examined there for the next 20 days. This means - no matter what, I have one visit per 20 days … Even if I am dying.’* (woman with asthma, hypertension, 405).

Several participants visited private hospitals or clinics, when unable to get their needs met in other settings, spending much of their income and incurring debt. Similar to the government facilities, they described only accessing services for ailments that were severe or urgent. In these cases, families specifically visited private facilities when their children had urgent needs (often ignoring their own ailments for long periods).

In sum, specific practices helped patients access care and have the confidence to manage their illness. However, a variety of factors influenced their ability to access high quality, comprehensive care, including the lack of secondary and tertiary services, disjointed service delivery across various institutions, medication limitations or specific shortages, an absence of financial support for transportation barriers and operational criteria for determining who qualifies for clinical services. Across the health sector – government, NGO and private – negative engagements with institutions risked discouraging people from further attempts to seek care, resulting in exacerbation of disease and secondary complications. People living with NCDs require care that is well coordinated and able to meet their diverse health needs. The fragmentation and complexity in government and NGO health systems means that healthcare for this population is not comprehensive or well-coordinated and thus not of high quality. This reality places refugees with NCDs at greater risk for increased morbidity and mortality.

### Coping with illness and barriers to care

In the face of challenges to psychosocial wellbeing and physical health, as well as difficulties accessing comprehensive care, Syrian refugees make a myriad of difficult daily decisions. Some of these decisions are about where to seek care, how often to do so, which alternatives to formal care to utilize and how to cope with financial limitations. These decisions and decision-making processes significantly impact overall health and wellbeing. Several participants highlighted a trend in care seeking behavior, specifically, utilizing private pharmacies as alternatives to seeking primary care. Taking on this behavior meant that patients were not being monitored by physicians and nurses and were instead making clinical adjustments (i.e., changing dosages, trying new medications) based upon their own understanding of their disease progression. The reasons for this practice varied and were described as follows,

*‘He was relieved by the injection [at the pharmacy]. But to tell you, this spring he experienced a relapse, so he is considering the injection again … What really stresses us is … you need to go there [to the clinic] at 6 in the morning to catch your turn otherwise you’re going to be late. You must be there early before the crowds come. You may need just a whole day before your turn. He goes ill and returns even more ill.’*

(man with asthma, 404)

*‘Then she said, “Where is she?”, I said, “She is at home, she is very old and she cannot come.” Then she said that she cannot give us the medicine until my wife comes. I told her, “Is that the procedure?” She said “Yes”, then I said, “You made up this procedure, I want to see the doctor …*” *I went out of the center and from that day, I did not come back for 2 years. Now I buy the medication myself, I have high blood pressure. My wife also has a high blood pressure. I go to the pharmacy and I buy the medication for me and for my wife.’* (woman with hypertension, 505).

In addition to relying solely on pharmacies for primary care, other forms of coping and decision-making occurred among participants. This included reducing medication dosages (without clinical consultation) in order to save money, begging strangers for money, selling food aid in order to purchase medications, as well as borrowing “similar” medications from neighbors and friends. Several participants illustrated these experiences,

*‘So, I sometimes save [money] by taking one tablet in one day, and nothing for two days … So that the packet is sufficient for a month … [My daughter] used to give me the medicine and ask me what I am taking. I used to tell her that it was fine and I can tolerate it. She told me that if I stop it, it might cause coagulation. But I can only depend on God.’* (man with hypertension, 621).

*‘My mother goes out and sorry to say that, but she begs [for] money and brings the medicine for me. I went once with her and saw people how kind they are with her, one dinar from here and two from there to bring the medicine. When I saw that I was touched, and asked her not to do it again. This is difficult for us because we were never like that and we never begged. She keeps telling me that people are kind and give me money, but I object to that … I am embarrassed. I am ashamed to tell you that my mother who is an old woman begs to bring me the medicine … We used to receive aide, but they stopped it … Now after they stopped the aide, my mother is forced to do [this].’* (woman with asthma, 413).

*‘We get our monthly coupon. For instance, we get two bags of milk. Instead of giving them to kids, we sell those bags and buy medication. We receive the UNICEF aid for [the] kids.’* (man with hypertension, 414).

*‘I got this [medication] from my neighbor when I ran out. I told her that I was dizzy and she told me to take a pill of this since it’s almost the same one, but this isn’t the same as I take. The one I take is foreign and costs four and a half dinars. It would last me for a month because I don’t take much from it.’* (woman with hypertension, 517).

In the face of rising healthcare costs, high transportation costs, long wait times in facilities and mobility difficulties, urban based refugees struggle to make decisions that support their health and wellness. Many participants described choosing pharmacies over clinics as their primary source of care and independently changing their medication dosage. A smaller number of participants reported raising funds or saving funds by begging on the street, selling food aid or borrowing a neighbor’s medication. These decisions are logical in the face of the current barriers. However, they may also result in numerous negative health consequences including a reduction in physician monitoring of NCD diagnoses, over-or-under use of medications and unsafe procedures in the pharmacy setting. Some of the personal attempts to raise funds were not only dangerous (i.e., prioritizing medications over food or begging from strangers), but also damaging to the dignity of those seeking care. In the long-term these behaviors risk exacerbating disease, increasing morbidity and mortality among members of the Syrian refugee community, and counteracting the efforts of healthcare providers. Other forms of coping were unrelated to access issues but instead served to reduce stress. One man with hypertension and diabetes shared, *‘I fear for myself, but I still smoke. Sometimes a cigarette is worth the whole world.’*

### Communities exhibiting solidarity through health

Refugees residing in urban settings and within host communities benefitted from the generosity of neighbors, grocery store owners, landlords and pharmacists. These community members often provided financial support, transportation for medical emergencies, free medications, discounted rent (or accepted receiving no rent for long periods) and many more offerings to support refugees in their community. Several participants expressed gratitude when describing how their neighbors helped with medications and physician visits.

*‘I have one [blood pressure monitor] that I borrowed from our Jordanian neighbor and I am embarrassed to keep going to him and now I have it with me. I see him every day in the mosque and I ask him if he wants it and he keeps telling me to keep it because I need it … I borrow it and he takes it back whenever he needs it.’ * (man with hypertension, 424).

*‘I told you that they only gave [medications] to me once or twice at [NGO 3], and then it was not available … Twice for the cortisone, as for the inhaler they told me to buy it from outside. I told them “from where can I bring them? I cannot afford it, I came to you.” They said they don’t know. My neighbors know that I have asthma. They are Jordanians and sometimes they give it to me because they have someone with asthma and they buy me one as well.’* (woman with asthma, 617).

*‘Interviewer: Do you measure it [blood pressure] at home? Respondent: No, I do [it] in my neighbor’s house.’* (woman with hypertension, 519).

In addition to specific health-related support, landlords were also helpful and reduced rents or accepted extremely delayed rental payments. This participant described the flexibility and generosity of his landlord, saying,

*‘We used to pay 220 dinars, now he takes 200 [for the rent]. Even if we are late - I did not pay since December - he does not force us to pay. Even if I do not pay until the next month, he does not say anything. The owner of the house is very nice to us … Right now, I have more than 150 debts in the grocery store, or 170. I am not sure. We pay later when the kids get paid - at the end of the month.’* (man with diabetes, hypertension, 512).

Shopkeepers or grocery store owners were also highlighted for their willingness to support refugee customers when they faced financial difficulties. Grocer relationships were specifically centered around employment or debt so customers could purchase food items. These gestures were a lifeline for many. One participant shared,

*‘Our neighbor has a store here that he lets me work in just so I can make ends meet. You’ve seen my son who is 14 years old? Sometimes he goes and sells coffee in the market … and he works in anything he can find. He isn’t allowed to work, but we are forced to send him to work. We pulled him out of school so he can help us.’* (man with asthma, 624).

The vast majority of participants described expressions of solidarity from their neighbors, landlords, shopkeepers and pharmacists. Solidarity manifested as drug donations from neighbors or pharmacists, decreased rent from landlords, credit from grocers and employment opportunities in family businesses. These gestures highlight that donating, bartering and borrowing is sustaining urban-based refugees, and in some cases, replacing formal aid structures with informal communal systems. The risk factors for noncommunicable diseases are also linked to informal communal systems. For example, decisions around food consumption, physical activity and smoking are frequently influenced by family, friends and social networks. Thus, efforts to address NCDs will benefit from more collective approaches that build upon kinship and strong social networks.

## Discussion

The study aim was to understand the depth and nuances of Syrian refugees’ experiences accessing NCD services in urban and semi-urban settings in Jordan. Four themes were prominent in the dataset and drew attention to opportunities for improvement in the humanitarian response for this community and for urban-based refugees regionally.

1. Emotional distress is a central concern and is frequently highlighted as the trigger for a non-communicable disease or its exacerbation.
2. Service provision across all sectors – government, NGO, private – is complex, inadequate, expensive and fragmented, making it physically and financially burdensome to engage with the health sector.
3. Given financial constraints, refugees make harmful health decisions in order to reduce financial burdens on themselves and their families.
4. Host-community members actively exhibit solidarity with their refugee neighbors and specifically do so during urgent or emergency health episodes.

The first theme prioritized mental health and psychosocial support (MHPSS) by identifying a strong link between mental and physical health and more specifically, the impact of mental health on one’s ability to control NCD symptoms and prevent secondary complications. The relationship between these variables was a central part of the vast majority of interviews and was identified as a barrier to wellness. The relationship between psychosocial wellbeing and physical health is well documented [22, 29–33]. Scott et al. [31] explored the development of chronic conditions in adult community members who had mental disorders, and found that having mental disorders was associated with the increased risk of developing chronic diseases. Verma et al. [29] noted that patients with diabetes were twice as likely to have depression in comparison to non-diabetic patients. Moussavi et al. [30] further studied depression as a comorbidity and discovered that those with an NCD and comorbid depression had the worst health scores, compared to those with only one NCD or only comorbid physical conditions.

Several psychosocial interventions have been explored, with mixed impact on NCD health outcomes. These include expressive writing for patients with asthma and poor lung function and mindfulness for stress reduction among people with diabetes and hypertension [34–36]. Nonetheless, there is strong evidence for an association between mental and physical health. These findings are relevant for humanitarians and health systems actors and require that (1) mental disorders be better recognized and integrated into primary care [37–39], (2) mental health programming be made culturally appropriate for affected populations [39, 40] and, 3 daily burdens (i.e., family separation, poverty, resettlement processes) be identified as mediators of the relationship between experiencing traumatic events and developing mental disorders [41, 42].

The second theme unearthed evidence that the health system does not provide comprehensive, coordinated, continuous, high-quality NCD care for refugee communities. NGOs were identified as central to primary care for refugees in Jordan, government facilities as only relevant for secondary/tertiary emergency care, and the private sector as a potential asset. Participants expressed physical exhaustion and financial burden associated with navigating services, usually visiting more than 3 facilities for care because of the narrow selection of services available at each institution. The Syrian refugee experiences examined in this study demonstrate the need to create a coordinated response between government, NGO and private sector actors through an expansion of the concept of a “health system.” Fostering high quality care for host-communities and refugees, alike, may reduce morbidity and mortality across the nation. Beaglehole et al. [43] in “Priority actions for the NCD crisis,” emphasized the need to strengthen health systems, consider new governance models, ensure drug availability and advance universal health coverage (UHC) to meet the needs of people living with NCDs. Globally, the UHC discussion must contend with the need for displaced persons to be covered under national insurance schemes. This discourse must also address the low levels of health spending and the resource limitations that exist in countries hosting the largest numbers of refugees [44]. One study among countries in the Association of Southeast Asian Nations (ASEAN) identified varying levels of government commitment to extending UHC to migrants, with Thailand exhibiting the most progressive policies – making coverage available for citizens, as well as regular and irregular migrants [45].

The third theme exposed that Syrian refugees were forced, by their circumstances, to use coping strategies that were dangerous and not conducive to good health. These included borrowing medications from neighbors, reducing medication dosages, using alternative therapies, begging strangers for money, selling food aid and incurring debt at private pharmacies. Participants were not only coping with their illness but coping with poverty in an expensive, urban environment where 80% of Syrian refugees live below the poverty line. Most Syrian refugees in urban settings in Jordan are unable to participate in formal work and are not covered by health insurance. This population relies on select NCD services being made free at NGOs, while government and private facilities provide care at a high cost. These dynamics are similar to that of other marginalized communities across the globe and place Syrian refugees at risk of catastrophic spending as a result of disease complications. These conditions are exacerbated by their protracted experience with displacement.

Dozens of studies in Egypt, West Bank and Gaza, Vietnam, India, Pakistan, Australia, Thailand, Tanzania and the United States have articulated that NCDs push vulnerable populations further into poverty through high out-of-pocket (OOP), catastrophic spending [46–48]. Eight studies from India focused on how diabetes influenced OOP spending among families and noted that OOP spending could be as high as 17.5% of household income [48]. One study in Egypt identified that 15.8% of Syrian refugees spent more than 30% of their non-food expenditures on healthcare [49]. Another study in the West Bank and Gaza noted that 12.5% of households were impoverished as a result of health spending in 2006 [50]. NCD diagnoses are specifically linked to catastrophic spending because they are often permanent diagnoses, require lifelong utilization of expensive medications and result in severe and expensive complications and hospitalizations when improperly controlled. These studies focus on a mix of low, middle and high-income countries but do not explore this issue in fragile or conflict-affected settings. It can be extrapolated, however, that the same financial burdens also impact forcibly displaced persons in low-and-middle income host countries. The coping mechanisms described in this study risk the health of affected persons and damages one’s sense of dignity. Solutions to catastrophic health spending center around UHC and should be prioritized as policymakers address the needs of refugee communities in urban, non-camp settings.

The final theme identified in this study is the value of strong host-community support to refugees in urban settings. The findings focused on the positive actions undertaken by host-community members, in support of Syrian neighbors, tenants and customers. The most frequent gestures included purchasing and/or sharing medications and providing transportation to the hospital for emergencies. One family stored the insulin of a Syrian neighbor because she did not have a refrigerator. Landlords and shopkeepers were also generous and frequently accepted lower rent and allowed Syrian customers to purchase groceries with agreements to pay at a later date. The literature on social cohesion in MENA is extensive and describes the tensions between Lebanese, Jordanian or Turkish hosts and Syrian refugees [51, 52]. Most tensions are related to perceptions about safety, employment and cultural influence. There are also concerns about service delivery and the sense that the influx has made it more difficult to access services including health [18]. The literature on solutions to social tensions in the region have been centered around youth, sport, livelihoods and education with limited attention to how health may support social cohesion goals [51, 52]. Health initiatives have the potential to play this role for host and refugee communities, specifically those with similar disease patterns. Innovation in health programming could support social cohesion efforts and build upon the goodwill that exists between hosts and new arrivals.

The findings from this study should be viewed in light of several limitations. First, we only explored three NCDs – diabetes, hypertension and asthma – and did not focus the in-depth interviews on other prevalent diagnoses. As a result, we cannot determine if other disease experiences are similar to those under study. However, we did recognize and document other common illnesses held by participants which are listed in the results section and incorporated into the analysis. We specifically learned about psychosocial distress, mental disorders and disc disorders and recognize the need for additional research on these topics. Second, this study is qualitative and identifies frequent uncontrolled illness among a population that is accessing care. The study would have benefitted from capturing clinical data such as blood pressure, blood glucose and lung function to determine the extent to which each participant’s illness was being controlled at the time of the interview. However, the study does document that many participants had uncontrolled disease as exhibited by self-reporting of their last laboratory test, frequent emergency department visits and the presence of secondary complications. Third, the study sample is narrow and was identified through review of IRC records. Future studies with this population should identify broader samples from across the nation. Even with these limitations, the findings of this study are relevant, timely and practical and unlike much of the empirical work on this subject, elevates the voices and perspectives of affected communities in order to identify effective program improvements and novel strategy and policy options.

## Conclusions

The aim of this study was to better understand the healthcare experiences of Syrian refugees with NCDs residing in urban Jordan. We sought to document refugee perspectives on their own health, experiences with health services, barriers to accessing care and methods for coping with illness and poverty. This objective was intentional because it is crucial that we learn from refugee voices, make improvements in service delivery and transform the humanitarian sector. Service users in fragmented systems are well placed to reflect on health access, health equity, system responsiveness, financial burdens and healthcare quality. Providers may also contribute to this learning, however, they often understand service delivery from their own organizational perspective and do not have the same exposure to the system’s complexity, as do users.

Results suggest various opportunities to influence host-country policy, enhance NGO service delivery, advance private sector partnerships, integrate mental health and psychosocial services into primary care, align health interventions with other sectors and leverage the solidarity exhibited by host communities. These findings provide practitioners and policymakers with entry points for delivering higher quality, more comprehensive NCD care and developing effective healthcare policy that meets the needs of all. Health services for refugees in urban settings have been restricted as a result of host-country limitations on access, NGO care that lacks collaboration across institutions, and weak private sector engagement. This restrictive approach is a deterrent to integration and an attack on the fundamental right to health. The SDGs and the third UN high-level meeting on NCDs invites health and humanitarian actors to recognize contemporary needs and respond with strong health systems that meet the needs of the most marginalized including those seeking refuge across international borders.

## Data Availability

Not applicable.

## Appendix

### Appendix 1: In-depth interview guide

In-depth interview guide: to understand refugee experience with health services.

Thank you for the opportunity to speak with you. The goal of this interview is to learn more about your experience with your health and health services in Jordan. By “health services” I mean, the services you receive from a physician or nurse at a clinic, hospital or pharmacy in Jordan. We will use the information to help agencies improve services. We will never share your name/identifying information with anyone outside of our research team.

Do you have any questions before we begin? Thank you. Let’s get started…

1. How do you feel about your health today?
  P: Do you feel in good health? Have you been diagnosed with any illnesses? What has it been like to live with [insert stated NCD]? What is your #1 concern about your health?
2. How do you get health services for your [insert stated NCD]?
  P: Where do you seek care for your [insert stated NCD]? What types of services have you received there?
3. Think of your last visit to [insert clinic/location]. Take your time. Walk me through what happened before your last visit [insert clinic/location].
4. Thank you for sharing. Now, can you tell me what happened on the day of the visit?
  P: What happened during the visit? Did you see a physician or a nurse? How did you feel about the visit? What went well during the visit? What could have been better during the visit? Were you prescribed any medications? Would you return to that place again if you needed help? Did you pay for services?
5. What advice did the physician or nurse give you about managing your [insert stated NCD]?
  P: What did she/he say about your diet? What did he/she say about exercise? Stress? or any other aspect of your personal life? What has it been like adhering to his/her recommendations? What would you need to be able to follow the physician’s recommendations?
6. Let’s talk a bit about your medications. What medications were your prescribed for your [insert stated NCD]? Tell me about your experience with getting and taking your medications.
  P: Tell me about the costs of your medications. How much of your monthly income goes to medication costs?
7. Other than [insert clinic/location from above], have you ever been to any other facilities/organizations that have helped you with your illness? How did they help?
  P: How did you find out about these organizations? Where are they located? What has been your experience with them? Did you have to pay for services?
8. Have you ever needed care but could not get it? Tell me about that experience.
  P: Do you go to anyone else for help with your [insert stated NCD]? Do you take anything else that is helpful to you? Do you go to an alternative place for help? How has your health changed?
9. We are almost done but I wanted to revisit something you said earlier… You mentioned…, can you tell me a bit more about that?
10. Is there anything I have not asked about that you would like to tell me?

## Abbreviations

ASEAN: Association of Southeast Asian Nations
IRC: International Rescue Committee
JHAS: Jordan Health Aid Society
LMIC: Low-and-middle income country
MENA: Middle East and North Africa
MHPSS: Mental health and psychosocial support
MSF: Médecins Sans Frontières
NCD: Non-communicable disease
NGO: Non-governmental organization
OOP: Out-of-pocket
PFA: Psychological First Aid
SDG: Sustainable development goals
UHC: Universal health coverage
